# Inconsistent Bodily Feedback? Interoceptive Sensibility Affects Internet Gaming Disorder in Emerging Adults

**DOI:** 10.3390/bs15070896

**Published:** 2025-06-30

**Authors:** Zhouchao Lv, Cuijing Li, Jiamiao Zhang, Jinbo He

**Affiliations:** 1 Key Laboratory of Adolescent Cyberpsychology and Behavior (CCNU), Ministry of Education, Wuhan 430079, China; lzc8023@ecut.edu.cn (Z.L.); lcj@mails.ccnu.edu.cn (C.L.); psyjiamiao@mails.ccnu.edu.cn (J.Z.); 2 Key Laboratory of Human Development and Mental Health of Hubei Province, School of Psychology, Central China Normal University, Wuhan 430079, China; 3 Student Counseling and Mental Health Center, East China University of Technology, Nanchang 330013, China; 4 Center for Mental Health, Guangxi Vocational College of Water Resources and Electric Power, Nanning 530023, China

**Keywords:** internet gaming disorder, interoceptive sensibility, positive outcome expectancy, flow experience, refusal self-efficacy

## Abstract

Internet gaming disorder (IGD) has been a prominent social problem throughout the world, causing various physical health issues, and interoceptive sensibility—the ability to perceive internal bodily signals—may be a key factor in this process. However, the relationship between interoceptive sensibility and IGD remains unclear. This study examined how interoceptive sensibility contributes to IGD, the potential mediating roles of positive outcome expectancy and flow experience, as well as the moderating role of refusal self-efficacy. The serial mediation and moderated mediation analyses of data collected from 1733 students (1031 males and 702 females, M_age_ = 19.56) revealed that interoceptive sensibility was positively associated with IGD, and this connection was serially mediated by positive outcome expectancy and flow experience. Moreover, refusal self-efficacy buffered the positive association between positive outcome expectancy and IGD and between flow experience and IGD. These findings suggest that interoceptive sensibility plays a crucial role in the occurrence of IGD, highlighting the importance of addressing bodily awareness in prevention and intervention strategies. Additionally, enhancing refusal self-efficacy may help mitigate the negative effects of positive outcome expectancy and flow experience, offering potential avenues for reducing IGD risk.

## 1. Introduction

Internet gaming disorder (IGD) is characterized by persistent, excessive gaming that impairs academic performance, relationships, physical health, and psychological well-being (Battle, 2013). It is especially prevalent among emerging adults, with prevalence rates reaching 4.7–5.6% in some populations (K. J. Chang et al., 2024; Mitchell et al., 2023). The biopsychosocial model of addiction posits that IGD is the product of the interplay between biological, psychological, and social factors (R. S. Chang et al., 2023; Skewes & Gonzalez, 2013; Sugaya et al., 2019). However, research has predominantly focused on psychological and social aspects, while physiological mechanisms remain relatively underexplored. In reality, gaming not only engages the brain’s reward system but also elicits a range of physiological responses, including increased heart rate and altered respiration patterns (Krarup & Krarup, 2020). These physiological changes may contribute to the addictive nature of gaming, yet they have been largely overlooked in current research.

According to Hebb’s optimal arousal theory (Hebb, 1955), individuals actively seek stimulation to maintain an optimal level of arousal, which is often activated through physiological responses during online gaming. These physiological changes induced by gaming may reinforce prolonged gaming behavior, therefore increasing the risk of addiction (Brady & Prentice, 2021; Vatsal et al., 2024). However, an individual’s ability to perceive and interpret these physiological changes—known as interoceptive sensibility—may play a crucial role in the occurrence and maintenance of IGD. Interoceptive sensibility refers to the subjective tendency to perceive and focus on internal bodily signals, including physiological cues such as breathing, heartbeat, and visceral sensations (Garfinkel et al., 2015). Research found that impairment in recognition of internal bodily signals may disrupt physiological and emotional regulation (Brewer et al., 2021) and promote gaming as a compensatory self-regulation strategy. Exploring interoceptive sensibility’s role in IGD could therefore provide valuable insights into underlying mechanisms and inform more effective prevention and treatment approaches.

### 1.1. Interoceptive Sensibility and IGD

Interoceptive sensibility’s abnormalities may contribute to addictive behaviors (Di Carlo et al., 2024; Forte et al., 2025; Hina & Aspell, 2019; Leganes-Fonteneau et al., 2022). Heightened interoceptive sensibility may increase individuals’ substance use and addictive behaviors by intensifying the perception of internal bodily states associated with such activities (Paulus & Stewart, 2014). For instance, studies indicate that increases in interoceptive sensibility promote alcohol consumption, which can modulate arousal states (e.g., emotional or somatic discomfort; Jakubczyk et al., 2019). Behavioral addiction studies have also found that individuals with gambling disorder display significantly heightened interoceptive sensibility (Smith et al., 2021; Wiśniewski et al., 2023). For instance, abnormally heightened sensitivity (e.g., excessive focus on cardiac arousal) may exacerbate anxiety, driving individuals to seek relief through addictive behaviors like gambling (London et al., 2024; Moccia et al., 2021). Conversely, some other studies found that diminished interoceptive sensibility may also be a risk factor for addictive behaviors (Costa, 2023; Di Carlo et al., 2024). For example, reduced interoceptive sensibility leads to a lack of awareness of internal signals and emotions (e.g., alexithymia; Di Carlo et al., 2024; Kahyacı Kılıç & Sönmez, 2024), which increases the possibility that individuals will use digital devices to regulate their emotional states. This heightens the risk of impulsive behaviors, such as internet and smartphone addiction (Costa, 2023; Di Carlo et al., 2024). However, there is currently no empirical research directly examining the relationship between interoceptive sensibility and IGD.

The Tripartite Neurocognitive Model of IGD illustrates that interoception plays a crucial role in the occurrence and maintenance of IGD (Wei et al., 2017). Specifically, dysregulated interoception, mediated by aberrant insular activity, transforms somatic and affective signals into compulsive gaming urges, compromises prefrontal inhibitory control, and shifts behavioral governance toward impulsive system dominance in individuals with IGD (Wei et al., 2017). Moreover, the activation of the insular cortex, a key brain region involved in processing interoceptive signals, is associated with enhanced reward system responses and weakened inhibitory control, leading to intense craving and difficulty in controlling impulses (Turel et al., 2021; Wei et al., 2017; J.-T. Zhang et al., 2016; Y. Zhang et al., 2016). Studies have found that individuals with heightened interoceptive sensibility are more likely to rely on emotions and physiological arousal, and they are better at transforming bodily sensations into positive emotional experiences, which influences their decision to engage in smartphone gaming (Y.-C. Chen et al., 2024). Thus, impairments in interoceptive sensibility may lead to heightened craving responses and compulsive gaming behaviors in individuals with IGD.

### 1.2. Mediating Role of Positive Outcome Expectancy

The outcome expectancy model suggests that addictive behaviors are driven by anticipations of positive outcomes, whether through pleasure or distress relief (Kouimtsidis et al., 2007). The positive outcome expectancy (POE) in IGD (i.e., the belief that gaming yields rewards such as emotional gratification, social status, or escapism; Wu et al., 2016) might constitute an important mechanism in the relationship between interoceptive sensibility and IGD. Individuals who have POE toward gaming may experience more positive feedback from gaming behaviors such as positive emotional experiences and physiological arousal (Perez & Katz, 2024). Studies indicate that POE positively impacts IGD, especially when they encounter frustration of psychological needs (Chamarro et al., 2020). Hou and Fang (2014) also found that individuals with IGD unconsciously associated internet games more closely with directly positive outcomes.

Further, interoceptive responses have been established to be closely related to POE. Based on the somatic marker theory of addiction, interoceptive responses can evoke emotional states and therefore influence individuals’ expectations of outcomes (Verdejo-García & Bechara, 2009). Empirical evidence on alcohol use behaviors indicates that enhanced interoception is positively correlated with positive moods and increases the expectation of positive outcomes (Leganes-Fonteneau et al., 2022). These interoceptive experiences can act as unconditioned stimuli, participating in associative learning processes by supporting the hedonic and incentive signature of alcohol effects, thereby further reinforcing positive future expectancies (Lovelock et al., 2021; Paulus et al., 2009). In addition, interoception affects an individual’s perception of value (Brewer et al., 2021). Heightened interoceptive sensibility can enhance the effects of substances on the body, boost the incentive value of addiction-related stimuli, and amplify the “wanting” system of addictive behaviors, thereby reinforcing positive outcome expectations for addictive behaviors (Pittenger & Bevins, 2013). Research revealed that an increase in interoceptive capacity significantly predicted increases in perceived stimulation effects as well as outcome expectancy (Leganes-Fonteneau, 2024; Marshall et al., 2019). Taken together, when people experience increased interoceptive sensations related to a particular activity (like gaming or drinking), they will perceive the activity as more appealing and become immersed in it, which can ultimately lead to addiction (Verdejo-García et al., 2012).

### 1.3. Mediating Role of Flow Experience

Flow experience refers to intense positive emotions derived from a sense of mastery and alignment with intrinsic goals, such as joy and satisfaction (Moneta, 2012). According to flow theory, flow is marked by a distortion of time perception, suppression of irrelevant stimuli, and a balance between control and challenge (Csikszentmihalyi, 2014), which are important characteristics in addiction. A substantial body of research has verified the positive relationship between flow experience and IGD (G. Zhang, 2015; D. Zhang et al., 2024). One possible explanation for this positive association is that flow experiences can distort time perception, leading to an underestimation of gaming session durations, and thereby increasing the risk of gaming addiction (Hull et al., 2013). In addition, flow experience, as a positive emotional reward, enhances players’ satisfaction and pleasure. This heightened reward value, coupled with impaired reward processing, lead to excessive gaming behaviors (Wang et al., 2024; Yao et al., 2022).

Flow experience, being an emotional experience related to bodily sensations, can also be influenced by interoceptive sensibility. This physiological mechanism may influence gaming behavior through two primary pathways. First, enhanced interoception improves individuals’ detection of immediate somatic signals, which facilitates intensified attentional focus, optimized emotional regulation, and reduced self-consciousness—essential prerequisites for achieving flow states (Day, 2020). Second, by minimizing cognitive resource allocation to environmental distractions, this mechanism promotes sustained task engagement while modulating somatic–emotional homeostasis, ultimately leading to highly focused immersive experiences (Day, 2020). Additionally, heightened interoceptive sensibility enhances trust and control over bodily signals, fostering deeper immersion in gaming behaviors (Cali et al., 2015). This dual reinforcement effect may facilitate the transition from flow experiences to pathological gaming behaviors through the restructuring of self-awareness frameworks and modifications in attentional allocation patterns (Kemp, 2020).

In addition, positive outcome expectancy (POE) may be closely associated with flow experience (T.-L. Huang et al., 2022; Rachmatullah et al., 2021). According to the expectancy-value theory, anticipation of rewarding outcomes can be viewed as the driving force of behavior (T.-L. Huang et al., 2024). Individuals who experience greater positive reinforcement from an activity are more likely to engage in it and subsequently enter a flow state (Moneta, 2012). In the context of online gaming, positive outcome expectancy, including anticipation of game-related rewards such as achievement, emotional regulation, or social feedback (Liao & Teng, 2017), can enhance engagement in gaming and lead to a highly immersive flow state. T.-L. Huang et al. (2022) investigated players’ game commitment in 1320 gamers. The results indicated that positive outcome expectancy from gaming (i.e., expectation of higher future skills) can immerse players in an in-game flow. The findings from Liao and Teng (2017) also revealed that the anticipation of increases in game levels enhances flow through perceived skill and challenge. These findings suggest that positive outcome expectancy is closely related to flow experience.

### 1.4. Moderation Effects of Refusal Self-Efficacy

Since the ability to resist gaming relies upon individuals’ self-efficacy to do so, self-efficacy may be a pivotal cognitive mechanism in the development and maintenance of IGD (Koh, 2016). Refusal self-efficacy in IGD is defined as the confidence to resist gaming despite internal triggers or external pressures (Bandura, 2012). Several studies have confirmed the protective role of refusal self-efficacy in addictive behaviors. For example, individuals with higher refusal self-efficacy will be less influenced by their peers’ drinking behaviors and consequently moderate their risk for higher levels of binge drinking (J. Chen et al., 2022). The risk-buffering hypothesis also emphasizes the role of refusal self-efficacy in attenuating the negative impacts of environmental risk factors on problem behaviors. Zhao et al. (2021) found that refusal self-efficacy can weaken the effects of risk factors (e.g., peer phubbing and boredom proneness) on smartphone addiction. Ehret et al. (2013) also validated the moderating effect of refusal self-efficacy and found that lower use of protective behavioral strategies only increases alcohol-related consequences in the past three months among individuals with lower refusal self-efficacy.

Interoceptive sensibility, which enhances awareness of internal bodily cues such as heart rate and stress, can increase the likelihood of gaming as a coping mechanism to manage emotional relief or escape. However, individuals with high refusal self-efficacy possess greater cognitive control, enabling them to resist the urge to game when these internal cues signal the need for emotional relief (Zhao et al., 2021). Furthermore, self-efficacy plays an essential role in motivating individuals to adopt and sustain healthy behaviors, and interventions that modify self-efficacy are effective in promoting health behavior change (Sheeran et al., 2016). Refusal self-efficacy protects against risky health behavior, such as substance abuse (Nyman et al., 2022). Refusal self-efficacy aids in engaging in healthier behaviors, such as physical activity or relaxation, instead of gaming in response to stress or anxiety (Hwajung, 2015).

Moreover, refusal self-efficacy helps individuals evaluate the short-term rewards of gaming against its potential long-term consequences, mitigating the influence of POE, which amplifies the perceived rewards of gaming (Stevens et al., 2016; Yang & Suh, 2017). This self-regulation weakens the automatic connection between expected rewards and gaming engagement, promoting healthier alternatives. Studies have shown that high refusal self-efficacy reduces the effect of POE on IGD (Koh, 2016). Additionally, refusal self-efficacy may play a protective role in buffering the impact of flow on IGD. On the one hand, refusal self-efficacy could enhance metacognitive awareness and reduce the attention bias associated with flow (Giardina et al., 2024).This allows individuals to assess their behaviors critically (Koyuncuoglu, 2023) and reorient toward alternative activities even in highly engaging activities like gaming (F. Li et al., 2023; Zhao et al., 2021). As a result, the flow experience in gaming is interrupted, and it ultimately prevents the automatic transition from immersion to addiction. Research has indicated that individuals with higher levels of refusal self-efficacy exhibit stronger self-monitoring abilities and maintain a degree of “vigilance” during immersion (Ayan & Topaloglu, 2017). This process prevents them from completely falling into automatic behavior patterns. On the other hand, individuals with higher refusal self-efficacy are more likely to set external control mechanisms, such as reminders or environmental cues, before engaging in gaming. These mechanisms can interrupt the chain of automatic behaviors during the flow state and thereby reduce the risk of uncontrolled gaming use (Cho, 2007).

### 1.5. The Current Study

**Figure 1:**
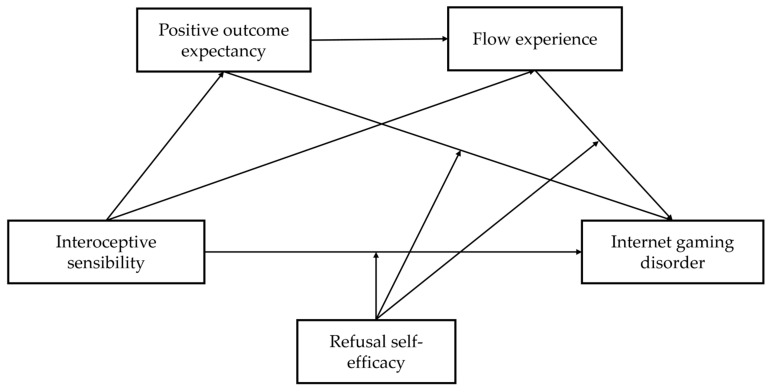
Hypothetical model.

Based on the literature reviewed above, the current study aimed to examine the unexplored relationship between interoceptive sensibility and IGD. Furthermore, we also explored the potential series mediating role of positive outcome expectancy and flow experience, as well as the moderating role of refusal self-efficacy in the association between interoceptive sensibility and IGD. The summary of all hypotheses is illustrated in Figure 1.

**Hypothesis** **1.** *Interoceptive sensibility is positively related to IGD*.

**Hypothesis** **2.** *Positive outcome expectancy mediates the positive association of interoceptive sensibility on IGD*.

**Hypothesis** **3.** *Flow experiences mediate the relationship between interoceptive sensibility and IGD*.

**Hypothesis** **4.** *Interoceptive sensibility positively predicts IGD through a chain mediation of positive outcome expectancy and flow experience*.

**Hypothesis** **5.** *Refusal self-efficacy moderates the positive impact of interoceptive sensibility on IGD*.

**Hypothesis** **6.** *Refusal self-efficacy moderates the positive predictive effect of positive outcome expectancy on IGD*.

**Hypothesis** **7.** *Refusal self-efficacy moderates the pathway from flow experiences to IGD*.

## 2. Materials and Methods

### 2.1. Participants and Procedures

This study adopted a cross-sectional, correlational design and used convenience sampling to recruit student populations in multiple provinces of China, including Jiangxi, Guangxi, and Hunan. Participants were recruited through announcements distributed across various schools and educational platforms. The study protocol was approved by the local ethics committee [approval number CCNU-IRB-202311007b] of a university in Wuhan, China. Informed consent was obtained from all participants. Data collection was anonymous, and only age and gender were recorded for statistical analysis.

After excluding participants who did not complete all the surveys, failed the attention checks, or reported never playing online games, the final sample consisted of 1733 mainly student participants aged from 13 to 30 years (M = 19.56, SD = 1.71; 59.49% male). These questionnaires took approximately 10 min to complete. Students who completed the survey received a participation certificate, which could be used to earn minor course credit. A complete overview of all sample demographics can be found in Table S1.

### 2.2. Measurements

#### 2.2.1. Interoceptive Sensibility

The Body Awareness Questionnaire (BAQ), an 18-item questionnaire developed by Shields et al. (1989), was revised by Luo (2023). The BAQ is widely used to measure interoceptive sensibility in individuals (Mehling et al., 2009). All items (e.g., “I notice that my body reacts differently to different foods”) were rated on a 7-point scale, with “1” indicating “very unlike me” and “7” indicating “very much like me”. The higher score indicates higher the subject’s interoceptive sensibility. The Cronbach’s α was 0.92 in this study.

#### 2.2.2. Positive Outcome Expectancy

A short version of the Positive Outcome Expectancy of Internet Gaming Questionnaire was used to measure positive outcome expectancy, and it was designed by Lin et al. (2008) and revised by D. Li et al. (2016). The scale consists of fifteen items (e.g., “Playing Internet games makes me feel happy”) and is rated on a 6-point Likert scale ranging from 1 (totally disagree) to 6 (totally agree). Higher scores represent a greater degree in positive outcome expectancy for internet gaming use. Great reliability and validity of this scale have been identified in Chinese college students (D. Li et al., 2016; Lin et al., 2018). The Cronbach’s α was 0.95 in this study.

#### 2.2.3. Flow Experience

Flow was assessed by the Questionnaire of Online Gaming Flow (H.-X. Zhang & Xie, 2008). Participants answered five items (e.g., “When playing online games, I would forget my surroundings”) on a 7-point scale, ranging from 1 (strongly disagree) to 7 (strongly agree). Responses across the items were summed, with higher scores indicating higher levels of flow during play. Existing studies have examined the reliability and validity of this questionnaire in Chinese college students (D. Zhang et al., 2024; H.-X. Zhang & Xie, 2008). The Cronbach’s α was 0.88 in this study.

#### 2.2.4. Refusal Self-Efficacy

The Online Game Refusal Self-efficacy Scale developed by C. Chen (2017) was used, containing three items, such as “I am confident in my ability to control my online game behavior”. A 7-point scale was used, with 1 representing “strongly disagree” and 7 representing “strongly agree”. The higher score indicates higher the degree of online game refusal self-efficacy. The Cronbach’s α was 0.93 in this study.

#### 2.2.5. Internet Gaming Disorder

The Internet Gaming Disorder Scale for College Students developed by Pontes et al. (2014) and revised by Qin et al. (2020) was used to measure IGD. The scale has 20 items divided into six dimensions: salience, emotion regulation, tolerance, withdrawal, conflict, and relapse. A 5-point Likert scale was used, with “1” indicating “strongly disagree” and “5” indicating “strongly agree”; a higher score indicates more serious online game addiction symptoms in the subject. The scale has been examined in college students (Qin et al., 2020), and the revised form has shown great reliability and validity in Chinese college students (C. Li et al., 2024). The Cronbach’s α was 0.94 in this study.

#### 2.2.6. Control Variables

Since gender, age, gaming status, daily gaming time, years of gaming, and gaming frequency are significantly correlated with IGD, these variables were included as covariates in the analysis to reduce their impact.

### 2.3. Data Analysis

All analyses were conducted using SPSS 23 statistical software. Prior to analysis, data were screened for basic statistical assumptions, including checks for normality (via skewness and kurtosis) and multicollinearity (using variance inflation factor and tolerance statistics). The PROCESS macro (model 6) of SPSS was used to examine the relationship between interoceptive sensibility and IGD and the (serial) mediating effects of positive outcome expectancy and flow. Model 89 of the PROCESS macro was selected to test the moderating effect of refusal self-efficacy. Indirect effects were examined using 5000 bias-corrected bootstrap samples with 95% confidence intervals (CIs). Significant interactions were further examined through simple slope analyses at ±1 standard deviation of the moderator.

## 3. Results

### 3.1. Descriptive Statistics and Correlation Analysis

The sample was tested for common method bias using Harman’s factor test, which showed that a total of eight factors had an eigenroot > 1, and the first factor had an explanatory rate of 27.08% (<40%) (Podsakoff et al., 2003). The highest absolute values of skewness and kurtosis were 0.68 (IGD) and 0.59 (flow), respectively, suggesting no serious deviation from normality. The variance inflation factor (VIF) was calculated for each predictor variable, with all VIF values below 10 (maximum = 1.469), indicating no significant multicollinearity issues. The tolerance values were all above 0.1 (minimum = 0.705), further confirming the absence of multicollinearity. Descriptive statistics and correlations were shown in Table 1.

### 3.2. Mediation Analyses

The mediation and moderated mediation analysis were analyzed after zero-mean normalization for interoceptive sensibility, positive outcome expectancy, flow experience, refusal self-efficacy, and IGD. Table 2 reports the results of multiple mediation analyses. interoceptive sensibility was positively associated with positive outcome expectancy (*B* = 0.27, *p* < 0.001) and flow (*B* = 0.10, *p* < 0.001) but had no direct effect on IGD (*B* = 0.01, *p* > 0.05). Positive outcome expectancy had a positive effect on IGD (*B* = 0.15, *p* < 0.001). Flow also had a positive effect on IGD (*B* = 0.21, *p* < 0.001). The indirect effects of interoceptive sensibility on IGD via positive outcome expectancy (*B* = 0.04, *SE* = 0.01, 95% *CI* [0.03, 0.06]) and flow (*B* = 0.02, *SE* = 0.01, 95% *CI* [0.01, 0.03]) were all significant. Moreover, positive outcome expectancy positively predicted flow (*B* = 0.39, *p* < 0.001). The serial indirect effect of interoceptive sensibility on IGD via positive outcome expectancy and flow was also significant (*B* = 0.02, *SE* = 0.00, 95% *CI* [0.015, 0.031]).

### 3.3. Moderated Mediation Analysis

**Figure 2:**
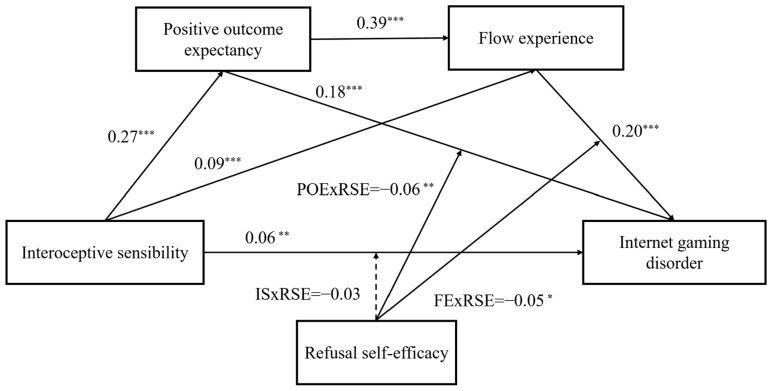
Serial mediating and moderating effects examined in this study. Path coefficients from interoceptive sensibility, positive outcome expectancy, and flow experience are from the serial mediation model. Path coefficients from refusal self-efficacy are from the moderated mediation model. * *p* < 0.05, ** *p* < 0.01, and *** *p* < 0.001.

**Figure 3:**
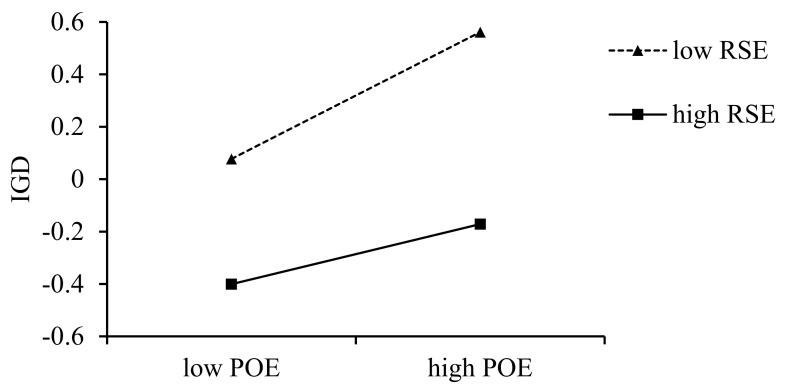
The moderating effect of refusal self-efficacy on the association between positive outcome expectancy and IGD.

**Figure 4:**
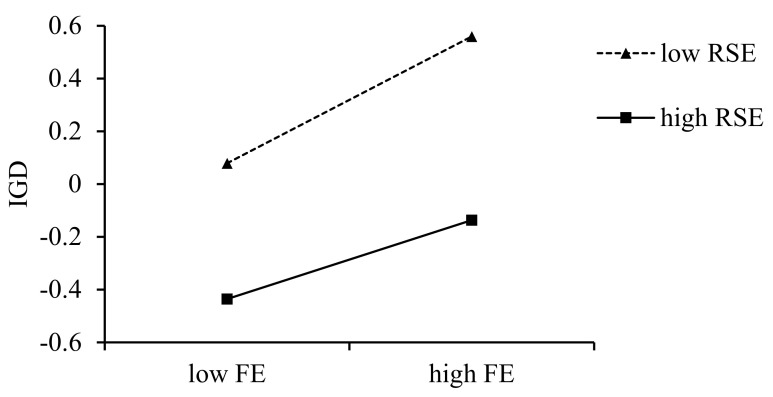
The moderating effect of refusal self-efficacy on the association between flow and IGD.

Model 89 of the SPSS PROCESS macro was used to test the moderating effects of refusal self-efficacy while controlling for the participants’ demographic variables. As shown in Table 3, interoceptive sensibility had a direct effect on IGD (*B* = 0.06, *p* < 0.01), but refusal self-efficacy cannot moderate the association between interoceptive sensibility and IGD (*B* = −0.03, *p* > 0.05). The moderating effect of refusal self-efficacy on the association between positive outcome expectancy and IGD was significant (*B* = −0.06, *p* < 0.01), as well as flow and IGD (*B* = −0.05, *p* < 0.05), with standardized coefficients displayed in Figure 2. As shown in Figure 3, the effect of positive outcome expectancy was lower when the level of refusal self-efficacy was high (*B* = 0.12, *SE* = 0.03, *t* = 3.49, *p* < 0.001, 95% *CI* [0.05, 0.18]) than when it was low (*B* = 0.24, *SE* = 0.036, *t* = 6.78, *p* < 0.001, 95% *CI* [0.17, 0.31]). As shown in Figure 4, the effect of flow was also lower when the level of refusal self-efficacy was high (*B* = 0.15, *SE* = 0.03, *t* = 4.42, *p* < 0.001, 95% *CI* [0.08, 0.22]) than when it was low (*B* = 0.24, *SE* = 0.04, *t* = 6.66, *p* < 0.001, 95% *CI* [0.17, 0.31]). 

## 4. Discussion

Exploring the relationship between interoceptive sensibility and IGD helps reveal the role of internal signal perception in addictive behaviors, enriches the understanding of the mechanisms underlying IGD, and provides a theoretical basis for the early identification, intervention, and prevention of IGD. The results of this study indicate that interoceptive sensibility is significantly positively correlated with IGD, which is consistent with previous research on substance addiction and gambling addiction (Jakubczyk et al., 2019, 2020; London et al., 2024). This association could be mediated by POE and flow experience. Refusal self-efficacy may be particularly important in reducing the association between POE, flow, and IGD. However, it did not significantly moderate the effect of interoceptive sensibility on IGD.

### 4.1. Association Between Interoceptive Sensibility and IGD

Our findings revealed a significant positive association between interoceptive sensibility and IGD. Individuals’ bodily feedback mechanisms may influence the onset and maintenance of IGD. Specifically, individuals with greater interoceptive sensibility were more confident in being able to recognize their own bodily sensations (Critchley & Garfinkel, 2017). A higher level of interoceptive sensibility was not conducive to individuals discontinuing gaming behaviors in a timely manner based on real bodily signals, nor did it help reduce the physical damage caused by excessive gaming. They may distract themselves from bodily sensations, further reinforcing a disconnection from the body and an avoidance of negative internal states (Di Carlo et al., 2024). This behavior may represent a maladaptive coping mechanism (Garfinkel & Critchley, 2013; Tsakiris & Critchley, 2016; Verdejo-García et al., 2012), where instead of addressing the discomfort, they “paralyze” themselves, using game as an escape or distraction from negative emotions or stress. Bodily sensations often provide key signals for understanding and coping with emotional states (Critchley & Garfinkel, 2017), but elevated interoceptive sensibility can create difficulties with self-regulation, discouraging control of impulsive behavior, which may lead to addiction (Di Carlo et al., 2024). Excessive interoceptive sensibility and possible body prediction errors can lead to imbalances in the interoceptive system, contributing to the maintenance of IGD.

### 4.2. The Mediating Role of Positive Outcome Expectancy and Flow Experience

As predicted, the mediating effect of POE on the association between interoceptive sensibility and IGD was significant. Interoceptive sensibility was associated with higher levels of outcome expectancy, which was consistent with the somatic marker theory of addiction. Physical and mental responses form “markers” of emotional states that are attached to expected outcomes (Verdejo-García & Bechara, 2009). Individuals with high level of interoceptive sensibility are likely to place greater trust in interoception and become more sensitized to rewards, leading to positive outcome expectations of “hedonic” pleasure. Moreover, over time, individuals become more sensitive to pleasurable expectations of addictive behaviors or substances during withdrawal or stress (Verdejo-García et al., 2012). Thus, interoceptive sensibility may increase positive expectations of gaming behaviors and may contribute to the development of IGD (D. Li et al., 2016; Lin et al., 2018; Wu et al., 2016).

Furthermore, interoceptive sensibility was also related to flow and was found to partially mediate the effect of IGD through flow experience. The association may result from interoceptive sensibility’s influence on attentional regulation, physiological arousal, emotional experience, and reward expectancy of flow. For instance, interoceptive sensibility can modulate an individual’s attention to focus on an online game (Paulus & Stewart, 2014), generating an attentional bias toward game cues and inducing gaming flow (Csikszentmihalyi, 2014; Rachmatullah et al., 2021; D. Zhang et al., 2024). This explanation is consistent with prior theoretical frameworks, though causality cannot be inferred due to the cross-sectional design. Individuals with higher levels of interoceptive sensibility are more attentive to internal body signals (Garfinkel et al., 2015) and are more likely to focus on perceiving internal body needs and behavioral motivations, such as responding more to cues for rewards (Paulus & Stewart, 2014) or generating pleasurable emotional experiences. The enjoyment and sense of achievement derived from flow experiences in gaming can strongly motivate individuals to repeatedly seek such states, which may increase the risk of gaming addiction (Footitt et al., 2024; Putra et al., 2024).

In addition, the findings of this study have shown that interoceptive sensibility had an indirect association with IGD via the serial mediating effect of POE and flow. This serial mediating model advances our understanding of how interoceptive sensibility is associated with IGD. That is, when an individual engages in gaming, they generate a series of internal body signals and tend to focus on the positive experiences and expected rewards, such as the pleasurable experience of addictive behaviors (Paulus & Stewart, 2014) and the reward expectations (Migliorini et al., 2013). This positive outcome expectancy triggers positive emotional experiences, reward anticipation and attentional bias, inducing gaming flow experiences and further leading to IGD (S. Huang & Zhu, 2021; Lai et al., 2021). Positive outcome expectations motivate individuals to engage in more gaming behaviors (Hou & Fang, 2014; Wu et al., 2016), and keep them “immersed” in the online game, focusing their attention on the game. Thus, POE associated with gaming positively predicts the experience of flow (Rachmatullah et al., 2021). The two can jointly induce individuals’ online gaming behaviors (D. Zhang et al., 2024).

### 4.3. The Moderating Role of Refusal Self-Efficacy

Refusal self-efficacy significantly buffered the effect of POE and flow on IGD, supporting the risk-buffering hypothesis (Luthar et al., 2015). Self-efficacy is an important variable in understanding why individuals change, maintain, and shape their behaviors, which directly influences their choice, effort, and persistence in behavioral activities, as well as the self-regulation of emotional states (Schunk & DiBenedetto, 2021). By enhancing individuals’ sense of control, it allows them to better assess the benefits and harms of gaming, focus on its negative consequences, and regulate their gaming motivations, reducing excessive gaming (Zhao et al., 2021). Additionally, high refusal self-efficacy mitigates the temptation of gaming flow, decreasing commitment and interest in prolonged gaming sessions (Greene et al., 2021; Lin et al., 2008). It helps individuals manage gaming time, set limits, and disengage when necessary, promoting positive emotional experiences and effective coping strategies (Du & Zhang, 2022; Miller et al., 2019). Thus, refusal self-efficacy serves as a protective factor against IGD by buffering both POE and flow effects.

Notably, no moderating effect of refusal self-efficacy was found in the association between interoceptive sensibility and IGD. This may be due to the fact that individuals with high interoceptive sensibility have a strong tendency to believe that physical signals from gaming are “positive“ (Garfinkel et al., 2015), or “gaming is not harmful to body”, while often ignoring undesirable physical reactions or disconnecting from actual physical feedback (Swinkels et al., 2021). Individuals with heightened interoceptive sensibility often overestimate their ability to discern bodily signals (Garfinkel et al., 2016), creating an illusion of confidence. The illusion of confidence can also lead individuals to feel greater self-efficacy (Bandura, 2012), encourage riskier behaviors, and foster unrealistic expectations, potentially masking underlying physical harm or self-doubt associated with gaming activities. It is crucial to recognize that even with high refusal self-efficacy, a disconnect between perceived capabilities and actual skills may hinder an individual’s disengagement from gaming.

Overall, these findings provided further support for theoretical models that emphasize interoception as an important mechanism for understanding IGD (Wei et al., 2017). They also support the theory of an imbalance in mind–body interactions among individuals with IGD. Interoceptive sensibility may explain both the reasons and mechanisms behind why individuals with IGD perceive contradictory internal body signals.

### 4.4. Implications and Limitations

This study is among the few that explore the link between interoception and behavioral addiction, confirming a positive correlation between interoceptive sensibility and IGD. It suggests that heightened interoceptive sensibility may contribute to IGD by disrupting internal self-regulation mechanisms. Therefore, preventive strategies targeting the effective management of interoceptive sensibility could help individuals better regulate their physiological and emotional responses to gaming-related stimuli, thereby reducing the risk of IGD. Approaches such as mindfulness training (e.g., Mindfulness-Based Stress Reduction) can cultivate non-judgmental awareness of present-moment bodily and emotional states, thereby reducing impulsive reliance on gaming (Bakhtiari et al., 2025; Lan et al., 2018; Paulus et al., 2013; Sancho et al., 2018). Physical exercise, including aerobic and resistance training, supports interoceptive functioning by activating relevant brain regions and promoting neurobiological changes that enhance emotional resilience and decision-making (Aryani et al., 2024; Paulus et al., 2013). Additionally, real-time biofeedback techniques, such as heart rate variability monitoring, help individuals visualize and regulate physiological responses during gaming urges (Rominger et al., 2021; Schillings et al., 2022). Together, these interventions offer promising pathways for preventing and alleviating IGD.

Despite these contributions, this study has several limitations that may influence the interpretation of results. First, the cross-sectional design of the current study precludes any causal inferences. Although the relationship between interoceptive sensibility, positive outcome expectancy, flow experience, refusal self-efficacy, and IGD has been confirmed by SEM analysis, further studies with longitudinal and experimental methods are needed to investigate the potential causal effects. Second, the use of the general population playing online games who volunteered to participant might generate a self-selection bias. This also limits the generalizability of the findings to broader, more diverse populations. Future studies could address this limitation by using more diverse and representative samples to improve the generalizability of the findings. Third, this study focused solely on interoceptive sensibility, without assessing interoceptive accuracy or awareness, thus offering only a partial view of the interoception–IGD relationship. Future research could expand on this by including measures of interoceptive accuracy and awareness to better understand the full relationship between the interoception and IGD.

## 5. Conclusions

Generally, this study verified the positive association between interoceptive sensibility and IGD, which was mediated by positive outcome expectancy and flow. Furthermore, refusal self-efficacy moderated the positive associations between positive outcome expectancy and IGD, and between flow and IGD, but it did not influence the effect of interoceptive sensibility on IGD. These findings underscore the practical applications and emphasize the importance of ongoing research and intervention strategies that address the impact of interoceptive sensibility on IGD.

## Figures and Tables

**Table 1:** Descriptive statistics and correlations of the measured variables.

	1	2	3	4	5
1. Interoceptive sensibility	1				
2. Positive outcome expectancy	0.31 ***	1			
3. Flow experience	0.25 ***	0.53 ***	1		
4. Refusal self-efficacy	0.29 ***	0.10 ***	0.01	1	
5. Internet gaming disorder	0.11 ***	0.33 ***	0.35 ***	−0.23 ***	1
Mean (SD)	71.04 (20.48)	49.56 (16.21)	16.82 (7.00)	13.87 (4.86)	45.09 (15.83)
Skewness	−0.45	−0.54	0.00	−0.48	0.68
Kurtosis	0.43	0.03	−0.59	−0.43	−0.04

*** *p* < 0.001.

**Table 2:** Bootstrap results for direct and indirect effects.

Path/Effect	Estimate	*SE*	95% *CI*
Path 1	0.04	0.01	(0.03, 0.06)
Path 2	0.02	0.01	(0.01, 0.03)
Path 3	0.02	0.00	(0.02, 0.03)
Total indirect effect	0.08	0.01	(0.06, 0.11)
Direct effect	0.01	0.02	(−0.04, 0.05)
Total effect	0.09	0.02	(0.05, 0.14)

Path 1. Interoceptive sensibility → positive outcome expectancy → IGD. Path 2. Interoceptive sensibility → flow experience→ IGD. Path 3. Interoceptive sensibility → positive outcome expectancy → flow experience → IGD. Gender, age, gaming status, daily gaming time, years of gaming, and gaming frequency were controlled.

**Table 3:** Test of moderated mediation.

	POE			Flow Experience			IGD		
	*B*	*SE*	*t*	*B*	*SE*	*t*	*B*	*SE*	*t*
Age	−0.03 *	0.01	−2.15	−0.06 **	0.01	−4.75	−0.04 ***	0.01	−3.33
Gender	−0.13 **	0.05	−2.81	0.13 **	0.04	2.91	−0.22 ***	0.05	−4.68
Play game	−0.27 ***	0.07	−3.99	−0.12	0.06	−1.95	0.02	0.07	0.22
Gaming years	0.05 **	0.02	3.17	0.10 ***	0.02	6.42	0.00	0.02	0.09
Gaming frequency	0.15 ***	0.02	7.79	0.08 ***	0.02	4.33	0.03	0.02	1.70
IS	0.27 ***	0.02	12.80	0.09 ***	0.02	4.67	0.06 **	0.02	2.65
POE				0.39 ***	0.02	17.68	0.18 ***	0.03	6.78
FE							0.20 ***	0.03	7.39
RSE							−0.30 ***	0.02	−13.06
IS × RSE							−0.03	0.02	−1.74
POE × RSE							−0.06 **	0.02	−2.90
FE × RSE							−0.05 *	0.02	−1.98
*R* ^2^	0.25 ***			0.37 ***			0.25 ***		
*F*	94.61			141.68			48.85		

IS = interoceptive sensibility; POE = positive outcome expectancy; FE = flow experience; RSE = refusal self-efficacy. * *p* < 0.05, ** *p* < 0.01, and *** *p* < 0.001.

## Data Availability

All data generated or analyzed during this study are included in this article and its online Supplementary Materials files. Further enquiries can be directed to the corresponding author, Jinbo He.

## Supplementary Materials

The following supporting information can be downloaded at: https://www.mdpi.com/article/10.3390/bs15070896/s1. Table S1: Descriptive characteristics of participants in the total sample. Table S2. The Body Awareness Questionnaire. Table S3. Positive Outcome Expectancy of Internet Gaming Questionnaire. Table S4. The Questionnaire of Online Gaming Flow. Table S5. The Online Game Refusal Self-efficacy Scale. Table S6. The Internet Gaming Disorder Test (IGD-20 Test).

## Abbreviations

The following abbreviations are used in this manuscript:

IGD	Internet gaming disorder
POE	Positive outcome expectancy
