# Supplementary material for: Inconsistent Bodily Feedback? Interoceptive Sensibility Affects Internet Gaming Disorder in Emerging Adults
Source: Behav Sci (Basel). 2025 Jun 30;15(7):896. doi: 10.3390/bs15070896 (PMC12292238; doi:10.3390/bs15070896)
Supplement: Supplementary file 1 [file behavsci-15-00896-s001.zip › behavsci-3604858-supplementary.pdf]

# Inconsistent bodily feedback? Interoceptive sensitivity affects internet gaming disorder in emerging adults

***Supplementary material***

Table S1. Descriptive characteristics of participants in the total sample.

| Variable | Total Sample (N = 1733) |
|---|---|
| **Age, *M (SD)*** | 19.56(1.71) |
| **Gender, *n* (%)** | |
| Male | 1031(59.5) |
| Female | 702(40.5%) |
| **School, *n* (%)** | |
| East China University of Technology | 866(50) |
| Shanxi vocational & technical college of finance & trade | 204(11.8) |
| Jiangxi Tourism and Commerce Vocational College | 202(11.7) |
| HeChi University | 162(9.3) |
| ShangRao Preschool Education College | 109(6.3) |
| Jiangxi Engineering Vocational College | 98(5.7) |
| Other | 92(5.3) |
| **Play internet games(whether), *n* (%)** | |
| Play | 1415(81.7) |
| Not play | 318(18.3) |
| **Gaming years, n (%)** | |
| Less than half a year | 413(23.8) |
| Half a year to 1 year | 188(10.8) |
| 1 year to 3 years | 310(17.9) |
| 3 years to 5 years | 338(19.5) |
| 5 years to 9 years | 277(16) |
| More than 9 years | 207(11.9) |
| **Gaming frequency, n (%)** | |
| Nearly don't play | 479(27.6) |
| Once per week | 251 (14.5) |
| 2–3 times per week | 448 (25.9) |
| 4–5 times per week | 259 (14.9) |
| Every day | 296(17.1) |
| **Duration of gaming per day, n (%)** | |
| Less than 1 h | 881 (50.8) |
| 1–3 h | 663 (38.3) |
| 3-5 h | 142(8.2) |
| 5+ hours | 47 (2.7) |

Table S2. The Body Awareness Questionnaire

| | Not at all | | | true about me | | | Very true |
|---|---|---|---|---|---|---|---|
| 1.I notice differences in the way my body reacts to various foods. | 1 | 2 | 3 | 4 | 5 | 6 | 7 |
| 2.I can always tell when I bump myself whether or not it will become a bruise. | 1 | 2 | 3 | 4 | 5 | 6 | 7 |
| 3.I always know when I've exerted myself to the point where I'll be sore the next day. | 1 | 2 | 3 | 4 | 5 | 6 | 7 |
| 4.I am always aware of changes in my energy level when I eat certain foods. | 1 | 2 | 3 | 4 | 5 | 6 | 7 |
| 5.I know in advance when I'm getting the flu. | 1 | 2 | 3 | 4 | 5 | 6 | 7 |
| 6.I know I'm running a fever without taking my temperature. | 1 | 2 | 3 | 4 | 5 | 6 | 7 |
| 7.I can distinguish between tiredness because of hunger and tiredness because of lack of sleep. | 1 | 2 | 3 | 4 | 5 | 6 | 7 |
| 8.I can accurately predict what time of day lack of sleep will catch up with me. | 1 | 2 | 3 | 4 | 5 | 6 | 7 |
| 9.I am aware of a cycle in my activity level throughout the day. | 1 | 2 | 3 | 4 | 5 | 6 | 7 |
| 10R.I *don't* notice seasonal rhythms and cycles in the way my body functions. | 1 | 2 | 3 | 4 | 5 | 6 | 7 |
| 11.As soon as I wake up in the morning, I know how much energy I'll have during the day. | 1 | 2 | 3 | 4 | 5 | 6 | 7 |
| 12.I can tell when I go to bed how well I will sleep that night. | 1 | 2 | 3 | 4 | 5 | 6 | 7 |
| 13.I notice distinct body reactions when I am fatigued. | 1 | 2 | 3 | 4 | 5 | 6 | 7 |
| 14.I notice specific body responses to changes in the weather. | 1 | 2 | 3 | 4 | 5 | 6 | 7 |
| 15.I can predict how much sleep I will need at night in order to wake up refreshed. | 1 | 2 | 3 | 4 | 5 | 6 | 7 |
| 16.When my exercise habits change, I can predict very accurately how that will affect my energy level. | 1 | 2 | 3 | 4 | 5 | 6 | 7 |
| 17.There seems to be a "best" time for me to go to sleep at night. | 1 | 2 | 3 | 4 | 5 | 6 | 7 |
| 18.I notice specific bodily reactions to being overhungry. | 1 | 2 | 3 | 4 | 5 | 6 | 7 |

Table S3. Positive Outcome Expectancy of Internet Gaming Questionnaire

| *Playing Internet games ...* | *Totally disagree* | *Often disagree* | *Sometimes disagree* | *Sometim es agree* | *Often agree* | *Totally agree* |
|---|---|---|---|---|---|---|
| 1. Makes one feel happy. | 1 | 2 | 3 | 4 | 5 | 6 |
| 2. Allows one to sell things and earn money. | 1 | 2 | 3 | 4 | 5 | 6 |
| 3. Allows one to express emotions. | 1 | 2 | 3 | 4 | 5 | 6 |
| 4. Allows one to pass away those boring hours. | 1 | 2 | 3 | 4 | 5 | 6 |
| 5. Allows one to have imagination. | 1 | 2 | 3 | 4 | 5 | 6 |
| 6. Allows one to have more topics to discuss with others. | 1 | 2 | 3 | 4 | 5 | 6 |
| 7. Makes one feel excited. | 1 | 2 | 3 | 4 | 5 | 6 |
| 8. Makes one's reactions quicker. | 1 | 2 | 3 | 4 | 5 | 6 |

| | | | | | | |
|---|---|---|---|---|---|---|
| 9. Makes one catch up with the trend. | 1 | 2 | 3 | 4 | 5 | 6 |
| 10. Reduces stress. | 1 | 2 | 3 | 4 | 5 | 6 |
| 11. Allows everyone to play games together. | 1 | 2 | 3 | 4 | 5 | 6 |
| 12. Allows one to feel a sense of achievement. | 1 | 2 | 3 | 4 | 5 | 6 |
| 13. Allows one to make more friends. | 1 | 2 | 3 | 4 | 5 | 6 |
| 14. Allows one to become detached with reality. | 1 | 2 | 3 | 4 | 5 | 6 |
| 15. Can train the flexibility of the fingers. | 1 | 2 | 3 | 4 | 5 | 6 |

Table S4. The Questionnaire of Online Gaming Flow

| | *Strongly disagree* | *Disagree* | *Sometimes disagree* | *Neither agree or disagree* | *Sometimes agree* | *Agree* | *Strongly agree* |
|---|---|---|---|---|---|---|---|
| 1. When I play online games, I forget my surroundings. | 1 | 2 | 3 | 4 | 5 | 6 | 7 |
| 2. I feel like I've come back from a journey to the real world after playing an online game. | 1 | 2 | 3 | 4 | 5 | 6 | 7 |
| 3. When I'm playing an online game, my body is in the room but my mind is in the gaming world. | 1 | 2 | 3 | 4 | 5 | 6 | 7 |
| 4. When playing online games, I feel flexible. | 1 | 2 | 3 | 4 | 5 | 6 | 7 |
| 5. I have great fun playing online games. | 1 | 2 | 3 | 4 | 5 | 6 | 7 |

Table S5. The Online Game Refusal Self-efficacy Scale

| | *Strongly disagree* | *Disagree* | *Sometimes disagree* | *Neither agree or disagree* | *Sometimes agree* | *Agree* | *Strongly agree* |
|---|---|---|---|---|---|---|---|
| 1.I feel confident in my ability to control my playing smartphone game behavior. | 1 | 2 | 3 | 4 | 5 | 6 | 7 |
| 2.I am sure I can control my playing behavior of smartphone games. | 1 | 2 | 3 | 4 | 5 | 6 | 7 |
| 3.I feel confident to control playing behavior of smartphone games. | 1 | 2 | 3 | 4 | 5 | 6 | 7 |

Table S6. The Internet Gaming Disorder Test (IGD-20 Test)

| | *Strongly disagree* | *Disagree* | *Neither agree or disagree* | *Agree* | *Strongly agree* |
|---|---|---|---|---|---|
| 1.I often lose sleep because of long gaming sessions. | 1 | 2 | 3 | 4 | 5 |
| 2R. I never play games in order to feel better. | 1 | 2 | 3 | 4 | 5 |
| 3. I have significantly increased the amount of time I play games over last year. | 1 | 2 | 3 | 4 | 5 |
| 4. When I am not gaming I feel more irritable. | 1 | 2 | 3 | 4 | 5 |
| 5. I have lost interest in other hobbies because of my gaming. | 1 | 2 | 3 | 4 | 5 |
| 6. I would like to cut down my gaming time but it's difficult to do. | 1 | 2 | 3 | 4 | 5 |
| 7. I usually think about my next gaming session when I am not playing | 1 | 2 | 3 | 4 | 5 |
| 8. I play games to help me cope with any bad feelings I might have. | 1 | 2 | 3 | 4 | 5 |
| 9. I need to spend increasing amounts of time engaged in playing games. | 1 | 2 | 3 | 4 | 5 |
| 10. I feel sad if I am not able to play games. | 1 | 2 | 3 | 4 | 5 |
| 11. I have lied to my family members because the amount of gaming I do. | 1 | 2 | 3 | 4 | 5 |
| 12. I do not think I could stop gaming. | 1 | 2 | 3 | 4 | 5 |
| 13. I think gaming has become the most time consuming activity in my life. | 1 | 2 | 3 | 4 | 5 |
| 14. I play games to forget about whatever's bothering me. | 1 | 2 | 3 | 4 | 5 |
| 15. I often think that a whole day is not enough to do everything I need to do in-game. | 1 | 2 | 3 | 4 | 5 |
| 16. I tend to get anxious if I can't play games for any reason. | | | | | |
| 17. I think my gaming has jeopardized the relationship with my partner. | | | | | |
| 18. I often try to play games less but find I cannot. | | | | | |
| 19R. I know my main daily activity (i.e., occupation, education, homemaker, etc.) has not been negatively affected by my gaming. | | | | | |
| 20. I believe my gaming is negatively impacting on important areas of my life.1. Makes one feel happy. | | | | | |
